# Hospitalization time is associated with weight gain in forensic mental health patients

**DOI:** 10.1192/j.eurpsy.2022.1535

**Published:** 2022-09-01

**Authors:** A.L. Pedersen, F. Gildberg, P. Hjorth, M. Højlund, K. Andersen

**Affiliations:** 1 University of Southern Denmark, Forensic Mental Health Research Unit, Middelfart, Denmark; 2 Region of Southern Denmark, Department Of Psychiatry Vejle, Vejle, Denmark; 3 University of Southern Denmark, Institute Of Public Health, Odense, Denmark; 4 Region of Southern Denmark, Department Of Mental Health Odense, odense, Denmark

**Keywords:** Forensic mental health patients, Body weight, Outpatients, Inpatients

## Abstract

**Introduction:**

Previous studies have found substantial weight gains in forensic mental health patients (FMHP) during hospitalisation. However, previous studies have not compared in- and outpatients, thus the weight change could be a general change over time. Research on the association between proportional hospitalization time (PHT) and weight change is lacking.

**Objectives:**

To investigate the association between time hospitalized and weight change among FMHP.

**Methods:**

Retrospective cohort study including FMHP with schizophrenia or bipolar disorder treated in the Region of Southern Denmark between 01jan2016 and 06apr2020. Patient characteristics and data on body weight was extracted from electronic medical records. The association between PHT and weight change per year was analyzed using linear regression. PHT was determined between each measurement as the total number of days hospitalized divided by the total number of days. Analyses were adjusted for gender, age, smoking, and antipsychotic medication.

**Results:**

The cohort included 328 FMHP, of which 91% were diagnosed with schizophrenia. PHT had a significant positive dose-response association with weight change, with an estimated difference of +4.0 kg/year for FMHP who were hospitalized 100% of the time, compared to FMHP who were exclusively treated as outpatients. The associations were different for FMHP belonging to different categories of BMI at baseline (test for interaction; p=0.006). Underweight hospitalized FMHP had the largest difference in weight gain compared to FMHP treated outside hospitals (+18.0 kg/year, p=0.006), and the difference was smallest in obese FMHP (+2.3 kg/year, p=0.21).

**Conclusions:**

PHT was positively associated with weight change among FMHP.

**Disclosure:**

No significant relationships.

